# The Ophthalmology Foundation launches global online examination for ophthalmologists in training

**Published:** 2025-01-31

**Figure:**
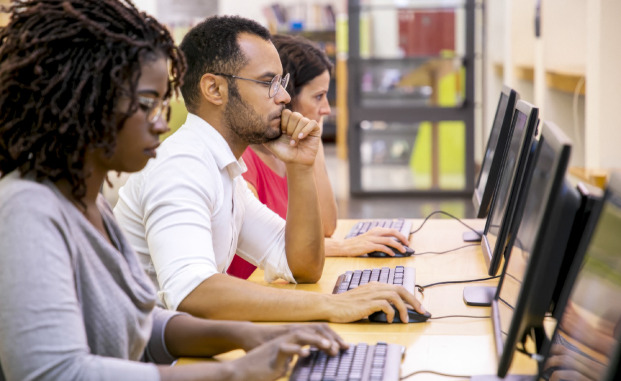
The exam is offered in English, Chinese, French, Portuguese, and Spanish.

© MANGOSTAR

The Ophthalmology Foundation has launched a groundbreaking international online examination designed for ophthalmologists in training, residents, and recently qualified ophthalmologists. Crafted to align with the highest standards and the professional requirements in the field. This initiative aims to standardize education, enhance clinical readiness, and set a high benchmark for ophthalmologists worldwide.

## A Global Assessment for Trainees

The examination allows trainees to evaluate their knowledge and clinical skills against international standards. Delivered online at local centers, it eliminates travel requirements, enabling participation from anywhere. Candidates who pass all three exams earn the post-nominal **FIOF** (Fellow of the International Ophthalmology Foundation), a mark of excellence that enhances their professional credentials. Additionally, passing the exam provides valuable points toward an **International Ophthalmological Fellowship Foundation (IOFF) Fellowship**, offering further career opportunities.

## Multilingual Access and Equitable Fees

The examination is offered in English, Chinese, French, Portuguese, and Spanish to accommodate diverse linguistic backgrounds. This ensures that candidates can take the test in their preferred language, reducing barriers to participation. Furthermore, the fee structure, based on the World Bank Index, adjusts costs according to income levels, making the exam more affordable for candidates in low- and middle-income countries. This commitment to equity ensures broader access to quality education and evaluation.

## Collaborations with Leading Institutions

The examination's development involved expertise from the Wills Eye Hospital and Wilmer Eye Institute, two of the world's leading centres for ophthalmology. Their input ensures the exam's content is rigorous and reflects the latest advancements in the field. Contributions from the Pan-American Association of Ophthalmology (PAAO) and the African Ophthalmology Council (AOC) provide additional regional insights, ensuring global relevance.

## Exam Format and Structure

The examination employs the “Angoff” method, a respected approach to determining passing scores based on question difficulty. This ensures fairness and consistency in evaluating candidates. The questions follow a single-best-answer multiple-choice format, emphasising clinical reasoning and decision-making.

The exam covers three critical areas of ophthalmology:

1. **Visual Science (120 questions):** This section evaluates knowledge of anatomy, physiology, pathology, and neuro-ophthalmology, essential for diagnosing and managing ocular conditions. Recommended to take during the first or second year of training.
2. **Optics, Refraction, and Instruments (60 questions):** Candidates are tested on optics principles, refraction techniques, and the use of tools like slit lamps and ophthalmoscopes. This section highlights the ability to measure and correct refractive errors accurately. Recommended to take second to fourth year of training.
3. **Clinical Ophthalmology (160 questions):** The largest section focuses on subspecialties, including retina, glaucoma, cornea, paediatric ophthalmology, and oculoplastic. This examination included clinical photographs, tables and videos. Recommended to take in the third to fifth year of training.

## Transforming Global Ophthalmology Training

The Ophthalmology Foundation's international examination sets a new standard for ophthalmology training and evaluation. By creating a globally recognised benchmark, it helps trainees assess their readiness for clinical practice while supporting institutions in identifying qualified professionals.

The examination's credibility is further enhanced by its partnerships with esteemed institutions, ensuring its alignment with global best practices. Its innovative features, including online accessibility, multilingual support, and equitable fees, reflect the Foundation's dedication to inclusivity and excellence.

With the introduction of this examination, the Ophthalmology Foundation continues to advance its mission of improving global access to high-quality eye care. By empowering the next generation of ophthalmologists, it is shaping the future of ophthalmology education and practice.

To register and learn more visit https://ophthalmologyfoundation.org/exams

The content of this page is supported by the Ophthalmology Foundation

**Example question**

*From Clinical Ophthalmology Question Bank*

**Figure:**
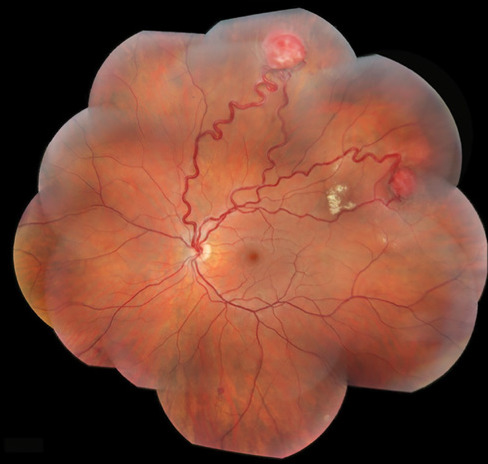
© DIDI Fabian


**1. The patient whose fundus is shown here is MOST likely to suffer from which of the following?**

- Optic pathway glioma
- Breast carcinoma
- Renal cell carcinoma T
- CNS medulloblastoma

